# First report of root-knot nematode, *Meloidogyne incognita* on calendula in Turkey

**DOI:** 10.21307/jofnem-2021-041

**Published:** 2021-04-01

**Authors:** Hürkan Ataş, Gülsüm Uysal, Çiğdem Gözel, Tevfik Özalp, Uğur Gözel, Zübeyir Devran

**Affiliations:** Department of Plant Protection, Faculty of Agriculture, University of Çanakkale Onsekiz Mart, Çanakkale, 17020, Turkey; Batı Akdeniz Agricultural Research Institute, Antalya, Turkey; Department of Plant Protection, Faculty of Agriculture, University of Akdeniz, Antalya, 07058, Turkey.

**Keywords:** *Calendula officinalis*, Identification, *Meloidogyne incognita*

## Abstract

*Calendula officinalis* L. (Asteraceae) is a traditional medicinal plant called pot marigold or English marigold. In this study, galled roots of pot marigold were collected from Balıkesir province of Turkey and egg masses were picked up from the roots of each plant with fine forceps. DNA was then extracted from samples and analyzed by species-specific primers referring to the most common *Meloidogyne* spp. Our results showed that *Meloidogyne incognita* was found as the only species in all the samples taken. The determination of *M. incognita* on calendula was done for the first time in Turkey.

Plant parasitic nematodes, especially root-knot nematodes (*Meloidogyne* spp.), cause serious yield losses in vegetables, ornamental and medicinal plants, and horticultural crops around the world (Devran et al., 2017; Karssen et al., 2013; Pintea et al., 2003). The plantation areas of ornamental and medicinal plants in tropical and sub-tropical countries have significantly increased recently in order to be used in pharmaceutical, perfumery, cosmetic and food industries (Pandey, 2017). *Calendula officinalis* L. (Asteraceae) has been planted as a traditional medicinal plant and is medicinally used in the world (Muley et al., 2009). Its extracts possess a wide range of pharmacological effects (Pintea et al., 2003). However, ornamental and medicinal plants can be attacked by several soil borne pathogens. Russo et al. (2008) pointed out that *C. officinalis* was infested with *Meloidogyne incognita* (Kofoid and White, 1919), Chitwood, 1949 in Italy. In another study, Brito et al. (2010) reported root-knot nematodes infected ornamental plants including calendula in Florida. Histopathological changes induced by *M. incognita* were investigated in five ornamental plants including *C. officinalis* and it was found moderately susceptible based on the galling index (Siddiqui et al., 2014). Similarly, Alijani et al. (2015) showed that growth factors of *C. officinalis* significantly decreased with increasing the number of *M. javanica* (Treub, 1885) Chitwood, 1949. To the best of our knowledge, in Turkey, the infestation of root-knot nematodes on calendula have not been reported.

We surveyed on calendula plants grown in open fields of Balıkesir province of Turkey, we observed symptoms of wilting in some calendula plants. When examining the roots of these plants, we observed galls on the roots (Fig. 1). Egg masses on the roots of calendula were collected using forceps and were put into incubation for hatching of second stage juveniles were fixed in TAF fixative and permanent preparations were made according to Seinhorst’s (1959) method. Measurements of approximately 25 J2s were made according to Karssen (2002) under Leica DM1000 stereomicroscope (Table 1). Adult females were removed from the roots of the calendula plants with a needle and scalpel under binoculars. Perineal patterns of the extracted females were cut in 45% lactic acid and their preparations were made in glycerin (Hooper, 1986). Morphological identification was made by us according to Jepson (1987) and Karssen (2002) (Fig. 2). The overall morphology and morphometric measurements of this population appear to be similar to *M. incognita* (Eisenback and Triantaphyllou, 1991; Whitehead, 1968). For molecular identification of *Meloidogyne* sp., genomic, DNA was isolated from J2s using the High Pure PCR Template Preparation Kit (Roche). Subsequently, DNA was analyzed by species-specific primers referring to common root-knot nematode species *M. incognita*, *M. javanica*, *M. arenaria* (Neal, 1889) Chitwood, 1949, *M. hapla* (Chitwood, 1949), *M. fallax* (Karssen, 1996), and *M. chitwoodi* (Golden et al., 1980) (Devran et al., 2018; Randig et al., 2002; Wishart et al., 2002; Zijlstra et al., 2000). PCR reactions were carried out in a SimpliAmp^TM^ Thermal Cycler (Applied Biosystems, CA, USA) according to Özalp et al. (2020). PCR products were run on a 2% agarose gel in 1X TAE buffer. Agarose gel was dyed with Xpert Green DNA Stain and viewed using the Gel iX Imager (Intas Science, Göttingen, Germany). *M. incognita*-specific inc-K14F/inc-K14R primers (Randig et al., 2002) and MincF1/MincR1 primer set (Devran et al., 2018) primers only produced an expected approximately 400 and 150 bp products, respectively, but other primers failed to amplify any products (Fig. 3).

**Figure 1:**
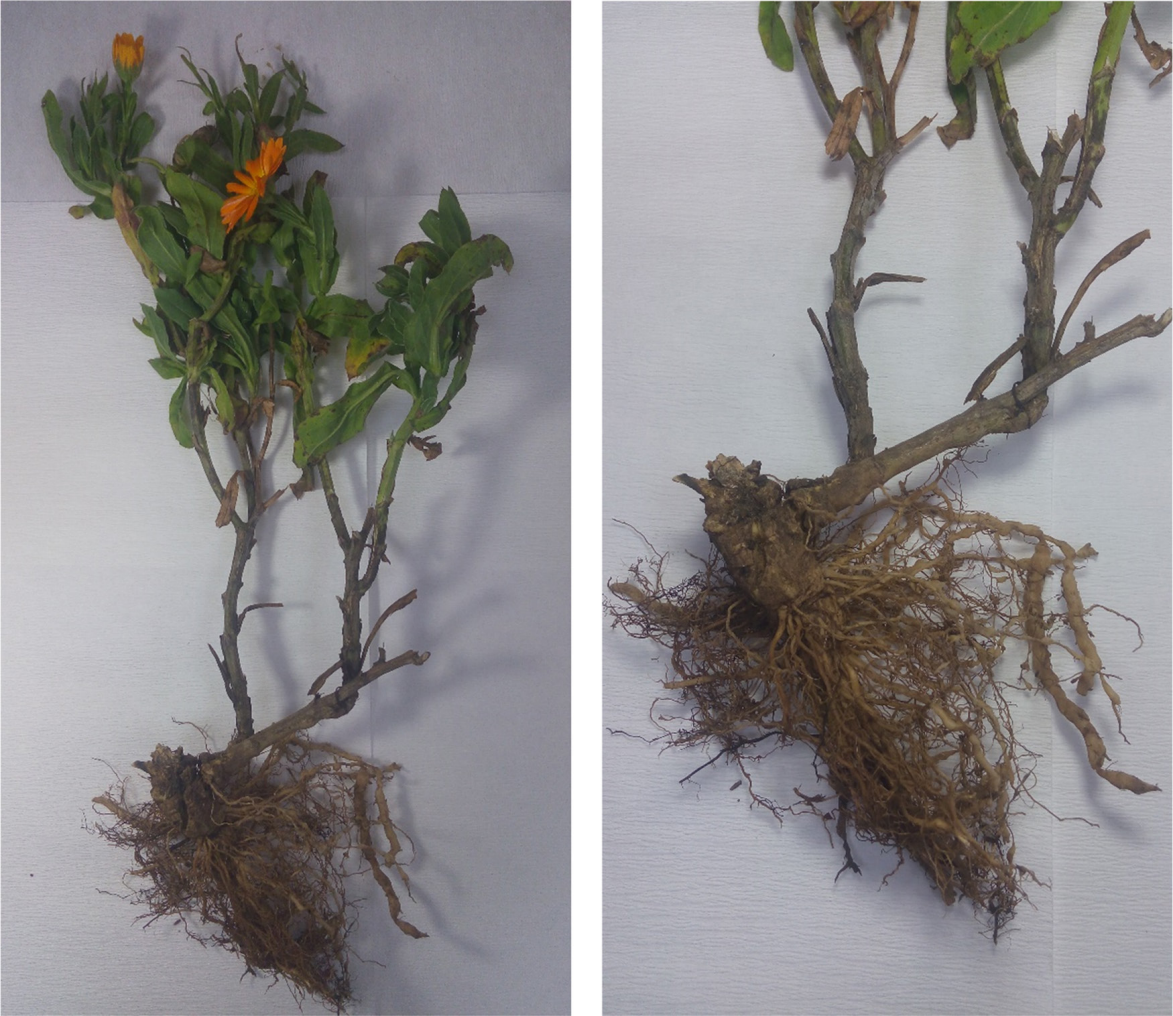
Galls caused by *Meloidogyne incognita* on the root of *Calendula officinalis* L.

**Figure 2:**
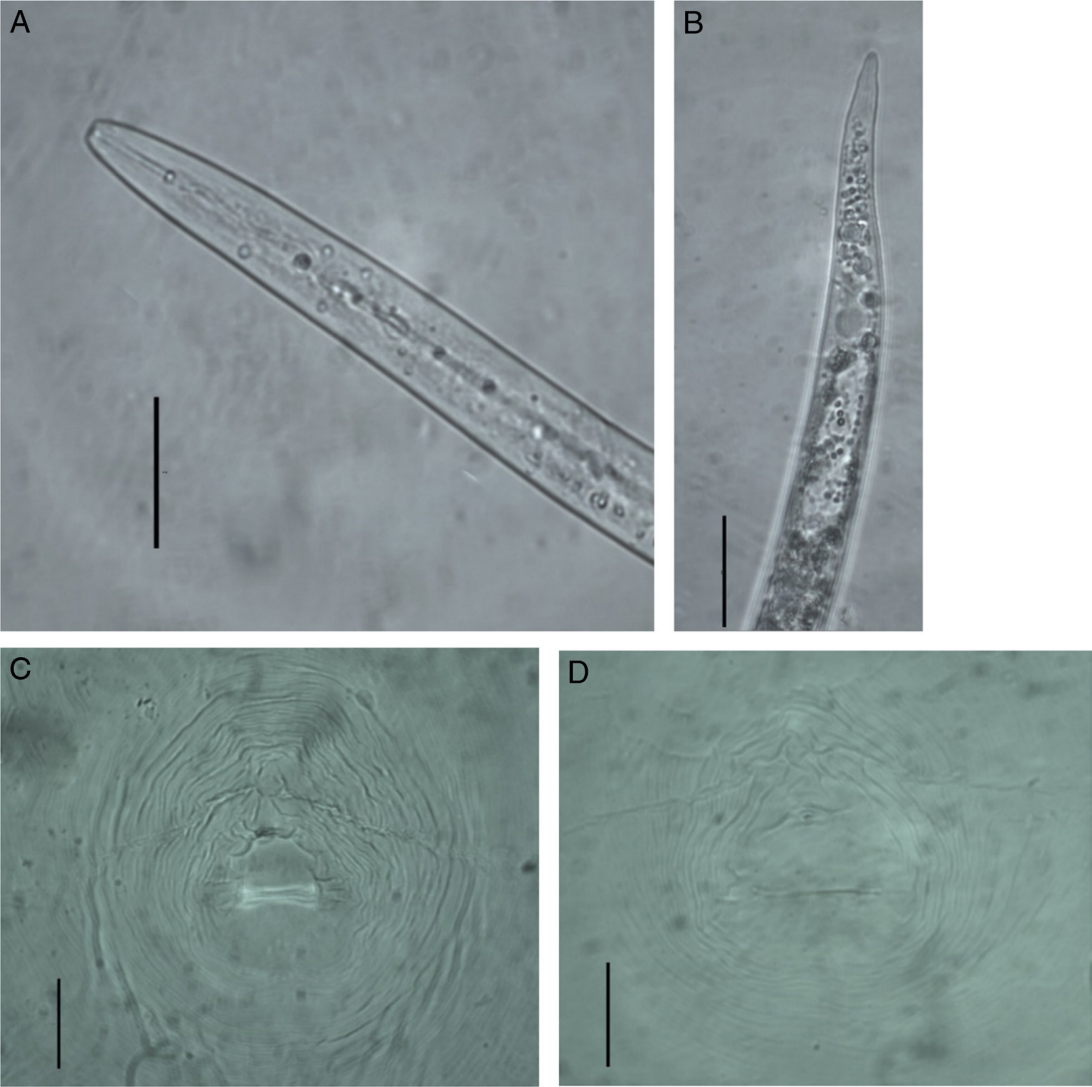
*Meloidogyne incognita* (Kofoid & White, 1919) from *Calendula officinalis* A: Anterior end region, B: Tail region, C-D: Perineal pattern (Scale bar: 20 µm).

**Table 1.** Morphometric characters of second-stage juveniles (J2) of *Meloidogyne incognita* on *Calendula officinalis.*

Diagnostic characters	This study	Whitehead (1968)	Eisenback and Triantaphyllou (1991)
Body length	399.79 ± 8.09 (389.27–418.95)	360–393	346–463
Body width	13.87 ± 0.86 (12.18–15.28)		
Body width at stylet base	8.98 ± 0.33 (7.96–9.60)		
Body width at anus	9.99 ± 0.68 (8.73–11.28)		
Stylet length	10.57 ± 0.66 (9.13–11.91)	10	10–12
DGO	3.02 ± 0.37 (2.30–3.94)	2–2.5	2–3
Tail length	44.93 ± 3.16 (39.15–52.00)		42–63
Excretory pore to head end	76.78 ± 5.59 (61.67–85.95)		
Body width at excretory pore	12.78 ± 0.56 (11.65–14.73)		
a	28.91 ± 1.57 (25.94–32.55)	29–33	
b	5.47 ± 0.29 (4.92–5.92)		
c	8.94 ± 0.64 (7.63–10.07)	8–9.4	
c’	4.51 ± 0.37 (3.87–5.31)		

Note: All measurements are in µm and in the form: mean ± s.d. (range).

**Figure 3:**
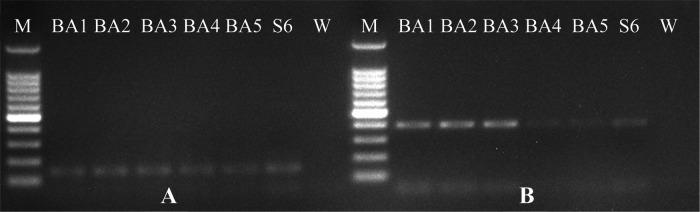
Amplified DNA of using *Meloidogyne incognita-*specific primers A: MincF1/MincR1 primer set, B: Inc-K14F/Inc-K14R primer set. M: DNA Ladder, Hibrigen 100 bp; BA1-BA5: Samples; S6: *M. incognita* (positive control); W: Water.

This is the first report identification of *M. incognita* on calendula in Turkey. It was also reported for the first time that plant parasitic nematodes on calendula in Turkey. The results demonstrated that calendula-growing areas in Balıkesir province were infested with *M. incognita.* These findings are important information for floriculturists and can be used to manage the damage caused by *M. incognita.*
